# Impact of Rural Dwelling on Emergency Department Visits for Individuals with Psychotic Disorders in Newfoundland and Labrador, Canada

**DOI:** 10.1093/schbul/sbaf198

**Published:** 2025-11-08

**Authors:** Zachary E M Giovannini-Green, Gerald Mugford, Zhiwei Gao

**Affiliations:** Division of Population Health and Applied Health Sciences, Faculty of Medicine, Memorial University of Newfoundland and Labrador, St. John’s, NL, A1B 3V6, Canada; Division of Population Health and Applied Health Sciences, Faculty of Medicine, Memorial University of Newfoundland and Labrador, St. John’s, NL, A1B 3V6, Canada; Division of Population Health and Applied Health Sciences, Faculty of Medicine, Memorial University of Newfoundland and Labrador, St. John’s, NL, A1B 3V6, Canada

**Keywords:** access to care, emergency medical services, rural health, schizophrenia, public health

## Abstract

**Background and hypothesis:**

Despite recognition that utilization of emergency departments (EDs) by youth with psychotic disorders is an area of concern, little research has been conducted investigating ED utilization in rural areas. This study tests the hypothesis: Is rural dwelling associated with increased utilization of EDs in youth with a psychotic disorder living in Newfoundland and Labrador (NL)?

**Study Design:**

Administrative databases were utilized for a retrospective cohort study. Patients diagnosed with a psychotic disorder between April 1, 2011, and March 31, 2022, between 15 and 24 years old, and who visited an ED at least once were included. Mean monthly visits measured ED utilization. Independent variables in the analyses consisted of age, sex, geographic region, and urban or rural residence. Linear regression modeling identified significant variables associated with mean monthly ED visits in the univariate and multivariate analyses.

**Study Results:**

Multivariate analysis indicated rural residence was significantly associated with an increased mean monthly number of ED visits compared to urban residence (B-Coefficient = 0.19, *P* < .01). Female sex was also significantly associated with increased mean monthly ED visits over male sex (B-Coefficient = 0.13, *P* < .01).

**Conclusions:**

This study identified rural residence as a factor that significantly increases the utilization of EDs by youth with psychotic disorders living in NL. However, further research is required to develop strategies to remove barriers to care and facilitate access for this population. This will be both financially beneficial for the healthcare system overall while also helping ensure the well-being of some of the most vulnerable people in our society.

## Introduction

Psychosis occurs as the presence of delusional beliefs, perceptual disturbances, or disorganized thoughts.^1^ Psychotic disorders are a cluster of conditions in which psychosis appears as a common symptom.^2^ The lifetime prevalence of psychotic disorders in the general population is approximately three percent.^3^ Research performed by de Oliveira and Tanner^4^ studying those with a psychotic disorder found that the first year after diagnosis cost hospitals an average of 24 000 US dollars. This does not include other forms of support, such as housing or employment. Nor does it include the indirect costs of psychotic illness, which are estimated to make up at least seventy percent of the total cost of the illness.^5^ Early intervention in psychosis is therefore a main area of research, given the ameliorating effects it has on illness outcomes.^6^^,^^7^ While the prevalence of diagnosed psychiatric conditions in youth is rising^8^ they are still unlikely to seek help.^9^ Self-stigma has been identified as a significant barrier that affects youths’ willingness to seek necessary supports,^6^ with only 20%-25% of youth requiring mental health supports seeking them out, and only half of those contacting healthcare providers directly.^9^^,^^10^ Social withdrawal, increased anxiety and irritability, as well as sudden preoccupation with bizarre thoughts, are symptoms that precede acute psychosis.^11^ These behaviors isolate vulnerable youth and can increase the risk of them leaving school^12^ and developing substance use disorders.^13^ This has been most recently brought to the attention of policymakers in Newfoundland and Labrador (NL) by the recent release of the Health Accord NL.^14^ This document clearly states that public engagement identified mental health as a serious concern for Newfoundlanders and Labradorians.

Treatment for psychotic illness is generally provided through early intervention services (EIS), which reduce the risk of mortality and improve outcomes, as well as showing a net benefit in terms of healthcare costs.^15^^,^^16^ However, challenges with EIS still exist. Bertulies-Esposito et al.^17^ identified that rural-based EIS in particular struggled with maintaining staff and serving a sparsely populated catchment area. This is consistent with other studies, which found that many services in urban areas were unavailable in rural areas, including access to regular and easy access to family physicians and specialists.^18^^,^^19^ While research has shown that individuals with psychotic disorders commonly access emergency departments (EDs),^20^ there is scarce research on how rural-dwelling individuals with psychotic disorders receive treatment. Furthermore, most available research in rural populations with psychotic disorders has been conducted in the global East and South,^21–23^ excluding a significant part of the global population. In the context of this study, NL has both a proportion of rural-dwelling individuals at twice the national figure^24^ and a rate of ED visits nearly twice the national average.^25^

The utilization of ED services for youth with psychotic disorders is an ongoing topic of research elsewhere in the world. However, little research has been done that specifically addresses the volume of ED utilization in rural areas in countries in the Western Hemisphere as compared to urban areas. This study will be undertaken to answer the following question: Is there a significant increase in the number of ED visits for youth diagnosed with a psychotic disorder living in rural vs urban areas in NL?

## Methods

### Study Design and Period

For this study, administrative databases from the Canadian province of NL were utilized to conduct a retrospective cohort study on data from April 1, 2011, to March 31, 2022. The data used in this study were obtained from Digital Health NL, the agency responsible for maintaining all health databases in the province.

### Study Population and Setting

To be included in the study, patients must have been diagnosed with a psychotic disorder between the ages of 15-24 in NL during the study period. Additionally, patients had to have had at least one visit to an ED in NL during the study period. Psychotic disorders included in the study were schizophrenia and schizoaffective disorder. Diagnoses were made in a hospital inpatient setting by a staff psychiatrist, and the date of first diagnosis was included in the administrative data captured. ICD-10-CA diagnostic codes were used to identify all patient diagnoses.

### Outcome Measures

For this study, the number of ED visits serves as the outcome variable. Mean monthly ED visits across the study period were defined as the total number of ED visits made by each individual divided by 132, the number of months in the study period. To achieve a full understanding of how individuals diagnosed with psychotic disorders utilize EDs in this province, all visits to an ED were included in this study, not only visits related to the individual’s psychotic disorder.

### Data Analysis

Independent variables included in the analyses consisted of the following: (1) age at diagnosis, (2) sex, (3) patient regional health authority (RHA), and (4) urban or rural residence as determined by Digital Health NL using the master geography file (MGF). Descriptive statistics were generated to describe the study population, including counts and proportions, and means and SD for categorical and continuous variables, respectively. Linear regression modeling identified the significant factors associated with the mean number of monthly visits to an ED in both the univariate and multivariate analyses. All analyses were conducted using SAS 9.4.

### Protocol

In response to a data access request by the authors, the following databases were accessed and linked by Digital Health NL:

1) The Provincial Discharge Abstract Database (PDAD) tracks the demographic, clinical, and administrative data of individuals when they are discharged from inpatient services or surgical day care services.
2) The provincial Client Registry (CR) collects demographic information from individuals presenting at a hospital or pharmacy as well as those applying for a Medical Care Plan (MCP) number.
3) The MGF uses the first three letters of an individual’s six-digit postal code and the Postal Code Conversion File (PCCF) to determine if an individual was living in an urban or rural area at the time of their first diagnosis. This method is also used to determine in which of the four RHAs an individual resides.
4) The MediTech Data – Emergency Department Module is an electronic patient database that collects demographic, clinical, and administrative data. Data collected includes date and time of arrival and discharge, as well as presenting complaint.

To link data from various databases, Digital Health NL created a unique de-identified patient number for each individual. Upon receiving the de-identified data, a single file was created that combined all relevant variables for the eligible individuals.

### Ethics Approval

This study has received ethical approval from the Health Research Ethics Board of the Health Research Ethics Authority of Newfoundland and Labrador (HREB #2022.214).

## Results

In the period from April 1, 2011, to March 31, 2022, 125 youth ages 15-24 were diagnosed with either schizophrenia or schizoaffective disorder. Of the 125 individuals in the study, all visited an ED at least once. Of the 125 individuals, the majority were male (75.2%), lived in an urban area (66.4%), and had Eastern Health as their RHA of residence (52.8%). The mean age at diagnosis was 20.64 years, and the mean monthly number of ED visits was 0.21 (see Table 1).

**Table 1:** Overview of Individuals in the Study

**Variable (*n* = 125)**	**Descriptives**
Sex	
Female	31 (24.8%)
Male	94 (75.2%)
RHA	
Central	8 (6.4%)
Western	25 (20.0%)
Labrador-Grenfell	26 (20.8%)
Eastern	66 (52.8%)
Urbanicity	
Rural	41 (32.8%)
Urban	83 (66.4%)
Age, mean (SD)	20.64 (2.40)
Mean ED Visits per Month, mean (SD)	0.21 (0.20)

Abbreviation: RHA, regional health authority.

In the univariate analysis, rural residence was found to be significantly associated with increased mean monthly ED visits (B-Coefficient = 0.13, *P* < .01). Female sex was also found to be significantly associated with increased mean monthly ED visits (B-Coefficient = 0.09, *P* = .03) (see Table 2).

**Table 2:** Univariate Analyses

**Variable**	**B-Coefficient**	**Standard error**	***P*-value**	**Type 3 *P*-value**
Sex				
Female	0.09	0.04	.03^a^	.03^a^
Male	0			
Age	0.01	0.01	.38	.38
RHA				.27
Central	−0.05	0.07	.51	
Western	<0.01	0.05	.97	
Labrador-Grenfell	0.08	0.05	.09	
Eastern	0			
Urbanicity				
Rural	0.13	0.04	<.01^a^	<.01^a^
Urban	0			

a indicates a statistically significant value

Abbreviation: RHA, regional health authority.

The multivariate regression indicated that individuals living in rural areas had significantly higher mean monthly ED visits than those living in urban areas (B-Coefficient = 0.19, *P* < .01). Females had significantly higher mean monthly ED visits than male patients (B-Coefficient = 0.13, *P* < .01) (see Table 3).

**Table 3:** Multivariate Analyses

**Variable**	**B-Coefficient**	**Standard error**	***P*-value**	**Type 3 *P*-value**
Sex				
Female	0.13	0.04	<.01^a^	<.01^a^
Male	0			
Age	0.01	0.01	.38	.38
RHA				.14
Central	−0.16	0.07	.03^a^	
Western	−0.04	0.04	.33	
Labrador-Grenfell	−0.08	0.53	.13	
Eastern	0			
Urbanicity				
Rural	0.19	0.04	<.01^a^	<.01^a^
Urban	0			

a indicates a statistically significant value

N.B.: No interaction was found between urbanicity and sex, age, or RHA and mean monthly emergency department visits.

Abbreviation: RHA, regional health authority.

## Discussion

### Main Findings of this Study

This study identified two demographic factors that are associated with increased mean monthly ED visits: rural residence and female sex. First, the B-coefficient in the multivariate regression indicated that rural mean monthly ED visits were 0.19 over urban mean monthly ED visits (Table 1). Rural-dwelling individuals live in relative isolation compared to urban-dwelling individuals and thus are not as able to access the same resources. This may be a factor contributing to their increased dependence on EDs to provide care, as seen in the literature.^18^^,^^19^ It should be noted that the urban–rural ratio found in this study population is consistent with the general population of the province.

Second, the B-coefficient for sex in the multivariate regression indicated that the mean monthly ED visits by females increased by 0.13 over male ED visits per month (Table 3), which is statistically significant (*P*-value < .01). This result indicates that further research is necessary to confirm the implications of this dissimilarity in ED utilization between males and females in other independent cohorts. Finally, it should be observed that all individuals who met the demographic and clinical criteria for the study also accessed an ED at least once during the study period. This further reinforces our understanding that those living with a psychotic disorder rely heavily on the services that EDs provide to them.

### What Is Already Known about this Topic?

The results of this study are consistent with those of Clark et al.,^19^ who found that the difference between urban and rural use of EDs produced a *P*-value of < .01. High rates of ED utilization in rural areas cause an economic burden on the healthcare system that can be reduced by conducting research in this area. In NL, the average number of visits made to EDs in 2018-2019 was nearly twice the average Canadian rate (922 vs 495 visits per 1000 people, respectively).^25^ Since the average cost of visiting an ED was estimated at $304,^25^ reducing this to the national average would make available a considerable amount of funds. These funds could then be used elsewhere to address the needs of those living in rural areas of the province, thus slowing or reversing the trend of rural EDs closing around the province.^26^

The second demographic factor identified that increased the mean monthly ED visits in the study population was female sex. Increased female utilization of EDs over males is consistent with the literature examining ED utilization for all complaints.^18^ Additionally, the literature indicates women are more likely than men to return to an ED shortly after treatment for psychotic symptoms. A Canadian study by Barbic et al.^27^ researched factors associated with a return to an ED within 30 days of being admitted and diagnosed with substance-induced psychosis. The authors found female sex was the most significant factor associated with a return to an ED within 30 days of a previous admission. However, the degree to which ED visits for females are higher than males in this study is not consistent with findings from other studies, being noticeably higher.

### Significance of this Study

The results of this study emphasize the need for a change in public health policy that increases support for youth with a psychotic disorder living in rural areas. The lack of community services compared to those available in urban areas necessitates that those living in rural areas access EDs to treat all symptoms of psychosis. Additionally, this research identifies a gap in services for females with a psychotic disorder. The authors of this study recommend policy changes that call for additional vital supports to be provided to rural areas to properly care for those living with psychotic disorders. The authors also recommend the addition of further training and support so ED staff are aware of the differences in symptom presentation between the sexes when treating psychotic disorders and can provide treatment appropriately. These recommendations offer a solution that would likely decrease the number of EDs visits that rural youth require to receive appropriate care. The increased ED utilization by rural-dwelling individuals with psychosis has yet to be translated into high-quality research examining this population in the Western hemisphere, but rather only in the global East and South.^21–23^ This gap in the literature is likely because urban residence has long been held as a risk factor for the development of psychosis and psychotic disorders.^28–30^ The authors hope that future research will continue to explore this vital topic.

### Limitations of this Study

This study was limited to those individuals who were diagnosed with schizophrenia or schizoaffective disorder during the study period. Individuals who were diagnosed before this period were not included in the study. Furthermore, only those aged 15-24 at the time of their diagnosis were included in this study. Individuals aged 15-24 constitute the demographic most affected by psychotic disorders, as shown by research linking earlier age of onset to poorer overall outcomes.^31^ Therefore, while research investigating individuals diagnosed after age 24 is a legitimate line of inquiry, the most vulnerable individuals affected by psychotic disorders were the focus of this study. Finally, the study population is from NL only, meaning caution should be used when generalizing specific results from this study to other regions.

## Conclusions

This study found an association between rural residence and ED utilization in youth with a psychotic disorder in NL. Rural residence was found to be associated with increased ED utilization over urban residence. Additionally, female sex was found to be associated with increased ED utilization over male sex. It is known that NL has an ED utilization that is nearly twice the national average.^25^ To improve health outcomes for this underserved and marginalized population worldwide, the authors recommend the implementation of changes in public health policy that facilitate equal access for all individuals, regardless of location. This will not only prove financially beneficial for the healthcare system but also help ensure the well-being of some of the most vulnerable people in our society.

## Data Availability

The data accessed to conduct this study are private health data held by the relevant health authorities. As such, the data cannot be made publicly available.
